# Molecular patterns of egyptian patients with non-squamous non-small-cell lung cancers: a clinicopathological study

**DOI:** 10.1186/s43046-023-00167-2

**Published:** 2023-04-03

**Authors:** Mohamed Said Ismail, Loay Kassem, Ahmed Al-Husseiny Ali, Fatma Elzahraa Ahmed, Mohamed Shalaby, Sally Magdy

**Affiliations:** 1 Department of Chest Diseases, Faculty of Medicine, Cairo University, Kasr Al-Ainy Hospital, Cairo, Egypt; 2 Clinical Oncology Department, Faculty of Medicine, Cairo University, Cairo, Egypt; 3 Surgical Oncology Department, National Cancer Institute, Cairo University, Cairo, Egypt

**Keywords:** Non-squamous non-small-cell lung cancer, Molecular profile, Epidermal growth factor receptor, Targeted therapy, Overall survival

## Abstract

**Background:**

Driver molecular aberrations, such as epidermal growth factor receptor (*EGFR*) mutation and anaplastic lymphoma kinase (*ALK*) gene rearrangement, play an important role in the oncogenesis and progression of non-squamous non-small-cell lung cancers (NSCLC). Therefore, this study aimed to detect the incidence of driver mutations among non-squamous NSCLC.

**Patients and methods:**

This was a retrospective-prospective cohort study on 131 patients with non-squamous NSCLC. Data on age, smoking status, chest symptoms, method of lung cancer diagnosis, molecular testing, including *EGFR* mutations in formalin-fixed paraffin-embedded (FFPE) tumor tissue and serum circulating tumor DNA using next-generation sequencing and *ALK* gene rearrangement by FFPE tumor tissue, and follow-up data regarding treatment modalities and outcomes were collected.

**Results:**

The median age of the patients was 57 years (range: 32–79 years). Out of 131 patients, 97 were males (74%), and 90 (68.7%) were smokers. Among 128 patients tested, 16 (12.5%) had *EGFR* mutations detected with either technique by formalin-fixed paraffin-embedded (FFPE) tumor tissue or/and serum circulating tumor DNA using next-generation sequencing, and 6 (4.7%) had *ALK* rearrangement by FFPE tumor tissue. The majority (62.6%) presented with metastatic disease. Among the 102 patients who received first-line systemic therapy, the objective response rate was 50.0% in mutated NSCLC versus 14.6% in non-mutated (*p* < 0.001). Among the eight mutated patients who received first-line tyrosine kinase inhibitors (TKIs), 7 patients achieved either complete response or partial response. Among the 22 mutated patients, the median overall survival was 3 months in those who did not receive targeted therapy versus not reached in those who received any type of targeted therapy (*p* < 0.001).

**Conclusion:**

Screening patients with newly diagnosed non-squamous NSCLC for driver mutations is essential for major prognostic and therapeutic implications. Early administration of TKIs in mutated patients significantly improves disease outcomes.

**Supplementary Information:**

The online version contains supplementary material available at 10.1186/s43046-023-00167-2.

## Background

Lung cancer is the leading cause of cancer deaths, accounting for 23.5% of all cancer deaths [1]. Most of the lung cancer cases (85%) are due to non-small-cell lung cancer (NSCLC), which exhibits a better prognosis than small-cell lung cancer (SCLC) [2]. In Western societies, nearly half of all patients are diagnosed with metastatic disease [1]. However, few data are available on the characteristics of Egyptian or Arab patients with NSCLC.

The incorporation of molecular profiling in NSCLC offers significant advancements in NSCLC care, particularly in the non-squamous subtypes. Non-squamous NSCLC, including Adenocarcinomas, Adenosquamous carcinomas, and large cell carcinomas, harbor several genetic aberrations. Roughly 60% of patients with lung adenocarcinoma are expected to have a driver mutation. Data on Caucasian patients show that the most common mutations are Ki-ras2 Kirsten rat sarcoma viral oncogene homolog (KRAS) (25%), epidermal growth factor receptor (EGFR) (15%), and anaplastic lymphoma kinase (ALK) (6%).

Racial discrepancies exist between the Asian and Caucasian populations, with significantly higher EGFR mutations in the Asian population [3], and deletion of exon 19 and L858R point mutation in exon 21 are the most common types detected in Asian patients [4]. Other demographic factors contributing to the differences noticed include gender with higher EGFR mutation rates in women and smoking history with more mutations in non-smokers. Regarding the histopathological subtype, more mutations are encountered in the adenocarcinoma subtype [5].

Identifying these genetic mutations in non-squamous NSCLC pathogenesis is essential for a better understanding of the disease [6] and permits further application of tyrosine kinase inhibitor (TKI) treatment that proved to have a higher objective response rate (ORR) and overall survival (OS) compared to standard chemotherapy [7].

This study aimed to detect the incidence of driver mutations among non-squamous NSCLC, clarify the outcomes in patients with mutated stage IV non-squamous NSCLC treated with targeted therapy in daily practice, and explore the association between targeted therapy and improved survival.

## Methods

### Study design

This was a single-center retrospective-prospective cohort study conducted at the Chest Department and Oncology Department, Kasr Al-Ainy Hospital, Cairo University, between January 2019 and May 2021. The study protocol was approved by the Institutional Review Board and Research Ethics Committee of Cairo University (approval no. MD-203–2019). All patients provided informed consent before the study procedures.

### Eligibility criteria

The study included patients older than 18 years with histologically proven non-squamous NSCLC. Patients with double primary cancer were excluded.

### Data collection

All patients were subjected to history taking, Eastern Cooperative Oncology Group performance status assessment, clinical examination, complete blood count, liver and kidney functions, and bleeding profile. Patients underwent chest contrast computed tomography (CT), abdominal imaging (CT or ultrasound), and brain imaging (CT or magnetic resonance imaging). Bone scan was performed if the patient had symptoms suggesting bone metastasis. Positron emission tomography-CT was performed at the treating physician’s discretion. Tissue biopsy was obtained via either bronchoscopy, imaging-guided biopsy, or surgical open lung biopsy. Samples were subjected to histopathological examination after staining with hematoxylin and eosin. Further immunohistochemical staining was performed at the treating physician’s discretion.

### EGFR testing

*EGFR* mutations (in exons 19, 20, and 21) were assessed using next-generation sequencing (NGS) in both the formalin-fixed paraffin-embedded (FFPE) tumor tissue and circulating tumor DNA (ctDNA). Testing in tumor tissue was performed using the therascreen EGFR RGQ PCR Kit for the detection of specific mutations (deletions and insertions) in the *EGFR* gene (29 somatic mutations including 3 insertions in exon 20 and 19 deletions in exon 19, S768I, L858R, L861Q, T790M, and G719X) in DNA derived from FFPE NSCLC tumor tissue using real-time polymerase chain reaction on the Rotor-Gene Q MDx 5plex HRM instrument [8].

Additionally, a liquid biopsy was obtained for ctDNA testing. Around 10 ml of peripheral venous blood was collected in Roche Cell-Free DNA Blood Collection Tubes (BCT) to stabilize cell-free total nucleic acids, and the sample was processed within 48 h, according to the Streck BCT protocol at Shanghai SmartQuerier Biomedicine Co. Ltd. Samples were centrifuged at 2,500 g for 10 min, and the collected serum supernatant was centrifuged at 16,000 g for 10 min, and both centrifugation processes occurred at 4 °C within 2 h from the collection. After collection of the resultant 3–5 mL of supernatant, the circulating free DNA extraction was performed using the QIAamp Circulating Nucleic Acid Kit (Therascreen, Qiagen Manchester Ltd) for NGS. For all patient blood samples, two NGS libraries involving plasma DNA and germline genomic DNA are required, with a minimum of 1 µg of germline DNA extracted from each sample [9].

### Testing for ALK rearrangement

*ALK* was tested on FFPE via VENTANA ALK (D5F3) CDx Assay, which is a rabbit monoclonal primary antibody that binds to the carboxyl terminus of human *ALK* in paraffin-embedded tissue sections stained with a BenchMark XT automated staining instrument. Then, the specific antibody was visualized using the OptiView DAB IHC Detection Kit, followed by the OptiView Amplification Kit and hematoxylin as counterstain. The OptiView DAB IHC Detection Kit is an indirect, biotin-free system for detecting mouse immunoglobulin G, mouse immunoglobulin M, and rabbit primary antibodies in sections of formalin-fixed, paraffin-embedded, and frozen tissue that are stained on the VENTANA automated slide stainers and then visualized by light microscopy [10].

Finally, and as per the updated testing guidelines for our center during the study conduction, few patients were additionally tested for *ROS1* rearrangement (25 patients) and programmed death-ligand 1 (PD-L1) expression (17 patients) by immunohistochemistry (IHC). ROS1 was screened by immunohistochemistry and confirmed by Reverse transcription of extracted RNA to c-DNA real time PCR Amplification kits for all fusion sites [E6; A19, E13; A20, E14ins11; del49A20, E2; A20, E18; A20&E17ins61, ins34A20], (Amoy Dx). PD-L1 was tested using the Dako 22C3 assay.

### Statistical analysis

Data management and analysis were performed using IBM SPSS Statistics for Windows, version 23.0 (IBM, Armonk, NY, USA). Numerical data were tested for normality by SHAPIRO–WILK test and presented as mean ± standard deviation or median and range. Categorical variables were compared using Chi-square and Fisher’s exact tests and presented as counts and percentages. Accuracy measures, including specificity, sensitivity, and predictive value of positive and negative tests, were used to assess the validity of serum *EGFR* testing versus FFPE tissue. OS was defined as the duration from histopathological diagnosis till death or last follow-up. Progression-free survival (PFS) was defined as the duration from histopathological diagnosis till cancer progression, death, or last follow-up. Patients without event at time of last follow up were censored. Both OS and PFS were estimated using the Kaplan–Meier method and compared using the log-rank test. A *p* value of 0.05 was considered significant.

## Results

### Patients’ characteristics

A total of 131 patients were included in our analysis. The median age was 57 years (range: 32–79 years), 97 were males (74%), and 90 patients (68.7%) were smokers with a median of 54 pack-years (range: 5–180 pack-years). The right lung was the primary tumor site in 77 patients (58.8%). All patients were diagnosed histologically with non-squamous NSCLC with at least a core biopsy (fine-needle aspiration cytology not accepted). Diagnostic biopsies were taken from the primary lesions in 103 patients (78.6%), metastatic lymph nodes in 10 patients (7.6%), and distant metastatic sites in 18 patients (17.6%). Most of the patients had adenocarcinoma (80.9%) with poorly differentiated tumors (54.2%). Table 1 shows the baseline characteristics of our cohort. Most patients presented with chest symptoms (cough, dyspnea, chest pain, etc.), whereas 13 presented with neurological manifestations diagnosed as brain metastases of NSCLC origin.

**Table 1:** Baseline characteristics of the study population

Patients’ characteristics		Number	%
*Age*	*Median (Range)*	*57 y*	*(32–79)*
Gender	*Male*	97	74%
	*Female*	34	26%
Smoking	*Smokers*	90	68.7%
	*Non – smokers*	41	31.3%
Histopathological subtype	*Adenocarcinoma*	106	80.9%
	*Large cell carcinoma*	20	15.3%
	*Adenosquamous*	4	3.1%
	*Muco-epidermoid carcinoma*	1	0.8%
Laterality of the disease	*Right lung*	77	58.8%
	*Left lung*	54	41.22%
Histopathological grade	*I*	2	1.5%
	*II*	57	43.9%
	*III*	71	54.6%
Stage at presentation	*I*	3	2.3%
	*II*	4	3.1%
	*III*	42	32.0%
	*IV*	82	62.6%

### Biological profile of our cohort

*EGFR* testing was performed on 128 patients of all stages using simultaneous testing on serum sample (ctDNA) and paraffin-embedded tumor tissue, and only three patients were not tested. Only 16 patients (12.5%) had *EGFR* mutations detected with either technique. *EGFR* mutations were slightly higher in females than in males (18.2% versus 10.5%) and in non-smokers than in smokers (17.5% versus 10.2%). Among those 16 patients, 15 tested positive on tissue block, whereas 1 had insufficient tissue for *EGFR* testing. Regarding liquid biopsy, only 12/16 patients (75.0%) tested positive for *EGFR* mutations (false negative: 25.0%). Table 2 shows the results of molecular testing in our cohort. Almost all patients with *EGFR* mutations (15/16) presented with stage IV disease (19% of all stage IV patients), whereas only one patient presented with stage III disease (2.3% of all stage III patients) (Table S2).

**Table 2:** Results of molecular testing in our cohort

Item		Number	%
Final EGFR result (including ctDNA and FFPE)	Positive	16	12.5%
	Negative	112	87.5%
	NA	3	
Exon involved in mutant EGFR	***19***	9	56.3%
	***L858R mutation in exon 21***	4	25%
	***20***	2	12.5%
	***18***	1	6.2%
ALK rearrangement	***Positive***	6	4.7%
	***Negative***	122	95.3%
	***NA***	3	
PDL1 expression	***Positive (*≥ *1%)***	9	52.9%
	***Negative***	8	47. 1%
	***NA***	114	

Similarly, 128 patients were tested for *ALK* rearrangement using IHC. Among them, six patients (4.7%) had *ALK* rearrangement mutations. Therefore, the total driver mutation rate of both EGFR and ALK was 17.2% (22/128).

Of those 6 *ALK*-positive patients, 4 (66.7%) were females, and 5 (83.3%) were non-smokers. *ROS1* rearrangement was tested in only 25 patients, and the results were negative in all of them. Finally, NSCLC patients with any positive targetable mutations (*EGFR* or *ALK*) were more common among non-smokers (30.0% versus 11.4%, *p* = 0.01) and more common in females (30.3% versus 12.6%, *p* = 0.02) (Table 3). Pooled targetable mutation status (*EGFR*/*ALK*/*ROS1*) was not significantly associated with the stage of NSCLC at presentation.

**Table 3:** Association between mutation status (EGFR and/or ALK) with gender and smoking status

Item		Any mutation (EGFR/ALK)				
		**Positive**		**Negative**		***P* value**
		**No**	**%**	**No**	**%**	
**Sex**	**male**	12	54.5%	83	78.3%	**0.02**^a^
	**female**	10	45.5%	23	21.7%	
**Cigarette smoking**	**yes**	10	45.5%	78	73.6%	**0.01**^a^
	**No**	12	54.5%	28	26.4%	

^a^Statistically significant

A total of 17 patients were tested for PD-L1 expression by IHC (Dako 22C3 test). Positive PD-L1 expression was detected in 9 patients (52.9%), whereas 8 patients (47%) had negative PD-L1 expression.

### Treatment modalities among patients with NSCLC

Surgical resection was performed in five patients with early disease (resection rate of 3.8%). Among the 44 patients with unresectable or inoperable disease (42 stage III and 2 stage II), 27 (61.4%) received chemoradiation, 16 (36.4%) received palliative systemic therapy, and 1 (2.3%) received best supportive care. One of the five patients who underwent surgery had positive ALK mutation and two of the 27 patients who received CCRT, had a mutation (one had an ALK mutation, and the other had EGFR mutation). Among the 82 patients with stage IV disease, 75 (91.5%) received palliative systemic therapy, and 7 (8.5%) received best supportive care.

A total of 102 patients received first-line systemic therapy (either *upfront therapy for* metastatic/inoperable disease or 11 patients who progressed after locoregional therapy). Of those patients, 94 received chemotherapy, whereas only 8 (6.1%) received a first-line TKI (two patients received crizotinib, four received gefitinib, one received osimertinib, and one received erlotinib). No patients received first-line immunotherapy.

Only 19 patients received second-line therapy: 6 (31.6%) received chemotherapy, 7 (36.4%) received a TKI (five received gefitinib and two received erlotinib), and 6 (31.6%) received immunotherapy. So, in conclusion, out of the 102 patients who received systemic therapy for advanced disease all of the 17 patients harboring a driver mutation received TKI therapy, eight of them as first line therapy, and seven as 2nd line therapy after progression on chemotherapy.

### Outcomes

Among the 102 patients who received first-line systemic therapy, 4 (3.9%) had complete remission, 18 (17.6%) had partial remission, 43 (42.2%) had stationary disease, and 37 (36.3%) had progressive disease. Regarding the biological subtypes, ORR was 10/20 (50.0%) in mutated NSCLC (either *ALK* or *EGFR*) versus 12/82 (14.6%) in non-mutated (*p* < 0.001). Among the eight mutated patients who received first-line TKIs, 7 (87.5%) achieved either complete or partial response.

**Fig. 1:**
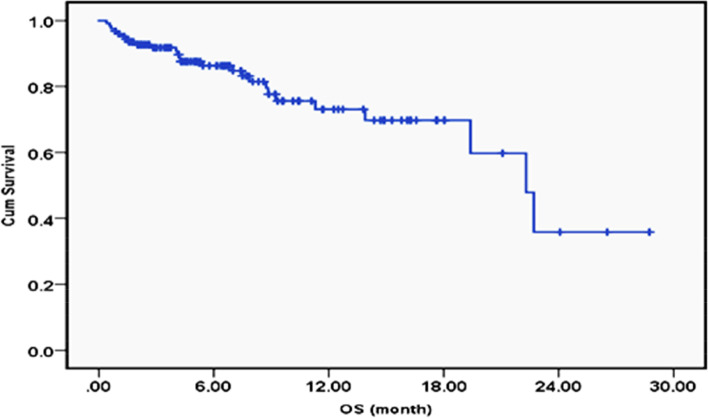
Kaplan Meier plot of OS for all patients

**Fig. 2:**
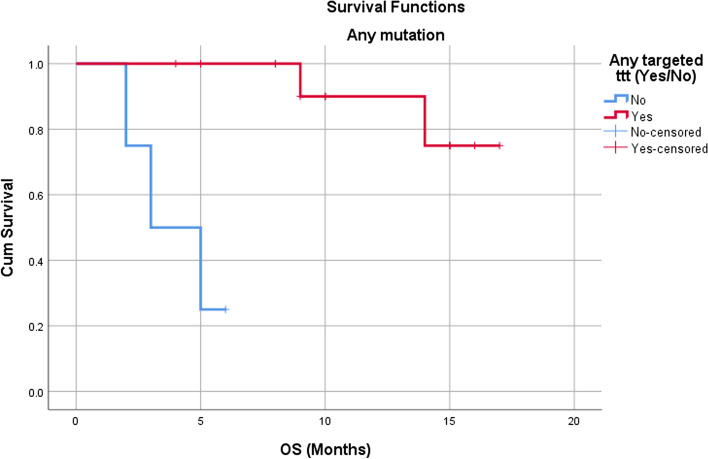
Kaplan Meier plot of OS among mutated NSCLC by targeted therapy received

After a median follow-up of 27 months (range: 5–30 months), OS rates were 86.4%, 73,1%, 69.7%, and 35.9% at 6, 12, 18, and 24 months, respectively the median OS for all patients was 22.3 months (Fig. 1). Among the 22 patients who had positive *EGFR* or *ALK* mutations, the median OS was 3 months in those who did not receive targeted therapy versus not reached in those who received any type of targeted therapy (*p* < 0.001) (Fig. 2).

## Discussion

To our knowledge, this study was one of the largest cohort studies of Egyptian and Middle Eastern populations conducted on 131 patients with non-squamous NSCLC to provide clinical and molecular characterization of this rapidly developing disease area. Most patients presented with locally advanced or metastatic disease, with only 3.6% could undergo surgical resection. *EGFR* and *ALK* were positive in 12.5% and 4.7% of patients, respectively. Not all mutant patients received targeted therapies, and only a minority received immune checkpoint inhibitors.

The majority of the study cohort were males (74%), with a male-to-female ratio of 2.8: 1. This ratio is higher than expected compared to studies from different ethnic backgrounds [9, 11]. This difference could have been even higher if our study included patients with squamous histology. Of note, 68.7% patients of our cohort were smokers, and only two females (5.9%) were smokers, which represents the social pattern of cigarette smoking in Egypt. Our population was relatively younger than those in similar cohort studies [11], with a median age of 57 years. A possible explanation may be the difference in the population pyramid distribution between Egypt and Western countries, with a lower life expectancy in the Egyptian population [12]**.**

This study sheds light on a very common problem faced in modern thoracic oncology, which is the adequacy of tissue biopsy for molecular testing. In this study, all patients were reviewed in a multidisciplinary meeting. This is an essential step in terms of acquiring enough tissue for molecular testing. In this study, in only three patients, we could not obtain enough tissue for *ALK*/*EGFR* testing. Bronchoscopic biopsy was possible in 66 patients (50.4%), denoting the feasibility of this modality even in non-squamous histology with a high incidence of peripheral tumors.

Interestingly, if the testing process was limited only to a population likely to be more positive (like females and/or non-smokers), around half of the mutant population would have been missed. This is of extreme importance considering the very high male-to-female ratio and smokers in our non-squamous NSCLC population. There are large racial discrepancies regarding *EGFR* mutation rates worldwide. In the Middle East and North Africa, the *EGFR* mutation rates in non-squamous NSCLC varied between 12.9% (22/170) in a Lebanese study [13] and 25.6% (42/164) in a multinational study including patients from Saudi Arabia, UAE, Qatar, Lebanon, and Algeria [14]. In this study, *EGFR* mutations were detected in 12.2% (16/128) of the patients and were much more common in those with stage IV disease than in those with stage I–III disease. Additionally, 4.6% patients of the tested population were positive for *ALK*. This is concordant with several studies worldwide [11, 15]. This makes the total driver mutation rate at 17.2% (22/128).

The National Comprehensive Cancer Network NSCLC Panel recommends testing molecular biomarkers, including *EGFR* mutations, *ALK*, *RET*, and *ROS1* rearrangements, PD-L1 expression, NTRK*1/2/3* gene fusions, *METex14* skipping mutations, and *KRAS* and v-Raf murine sarcoma viral oncogene homolog B (*BRAF)* mutations, for patients with NSCLC before initial treatment [16]. Unfortunately, broad molecular profiling was not reimbursed in Egypt at the time of our study. Recently, PD-L1 and *ROS1* testing have been included in the reimbursed panel.

Of note, patients with positive *EGFR*/*ALK* who did not receive a targeted therapy had poor outcomes with a median OS of 3 months. As expected, this was significantly worse than those who received any TKI during the therapy course (median: not reached, *p* < 0.001). This is consistent with almost every similar study denoting the importance of offering mutation-guided therapy as early as possible during the disease course [17–19]. Subsequently, the lack of molecular testing in stage IV non-squamous NSCLC may be associated with a shorter OS.

This study has some limitations due to its retrospective nature and the relatively heterogeneous population in terms of treatment strategies. A larger prospective registry is needed to provide clearer results on the treatment outcomes of Egyptian patients.

## Conclusion

This study describes in detail the demographic, anatomical, and molecular profile of Egyptian non-squamous NSCLC. Additionally, we shed light on the importance of expanding molecular testing in this cohort of patients. In patients with advanced NSCLC, early identification of targetable genomic aberrations and the introduction of targeted therapies and immunotherapy guided by the molecular profile can improve the disease outcome even in limited resources setting.

## Supplementary Information

**Additional file 1: Table: **Showed sensitivity, specificity, positive, negative predictive value and Accuracy of serum EGFR. **Figure: **Circle A showed EGFR detection by serum sample, circle B showed EGFR detection by paraffin block.

## Data Availability

All data generated or analyzed during this study are included in this published article.

## Abbreviations

ALK: Anaplastic lymphoma kinase
BRAF: B-Raf proto-oncogene
ctDNA: Circulating tumor DNA
EGFR: Epidermal growth factor receptor
KRAS: V-Ki-ras2 Kristen rat sarcoma viral oncogene homolog
LC: Lung cancer
NSCLC: Non-small cell lung cancer
OS: Overall survival
PDL1: Programmed-death ligand 1
ROS1: ROS Proto-Oncogene 1
SCLC: Small cell lung cancer
TKIs: Tyrosine kinase inhibitors
