# Supplementary material for: Molecular patterns of egyptian patients with non-squamous non-small-cell lung cancers: a clinicopathological study
Source: J Egypt Natl Canc Inst. 2023 Apr 3;35:7. doi: 10.1186/s43046-023-00167-2 (PMC13313946; doi:10.1186/s43046-023-00167-2)
Supplement: Supplementary file 1 — Additional file 1: Table: Showed sensitivity, specificity, positive, negative predictive value and Accuracy of serum EGFR. Figure: Circle A showed EGFR detection by serum sample, circle B showed EGFR detection by paraffin block. [file 43046_2023_167_MOESM1_ESM.docx]

**Table: Showed sensitivity, specificity, positive, negative predictive value and Accuracy of serum EGFR.**

| Statistic | **Value** | **95% CI** |
| --- | --- | --- |
| Sensitivity | 75.00% | 47.62% to 92.73% |
| Specificity | 100.00% | 96.76% to 100.00% |
| Positive Predictive Value | 100.00% |  |
| Negative Predictive Value | 96.55% | 92.30% to 98.49% |
| Accuracy | 96.88% | 92.19% to 99.14% |

**Figure:** Circle **A** showed EGFR detection by serum sample, circle **B** showed EGFR detection by paraffin block.

**B**

**A**

**4**

**1**
